# *Aucklandia lappa* Causes Membrane Permeation of *Candida albicans*

**DOI:** 10.4014/jmb.2009.09044

**Published:** 2020-11-04

**Authors:** Heung-Shick Lee, Younhee Kim

**Affiliations:** 1 Department of Biotechnology and Bioinformatics, Korea University, Sejongsi 3009, Republic of Korea; 2 Department of Korean Medicine, Semyung University, Jecheon 7136, Republic of Korea

**Keywords:** *Aucklandia lappa*, *Candida albicans*, cell membrane, DPH, permeability, ethidium bromide

## Abstract

*Candida albicans* is a major fungal pathogen in humans. In our previous study, we reported that an ethanol extract from *Aucklandia lappa* weakens *C. albicans* cell wall by inhibiting synthesis or assembly of both (1,3)-β-D-glucan polymers and chitin. In the current study, we found that the extract is involved in permeabilization of *C. albicans* cell membranes. While uptake of ethidium bromide (EtBr) was 3.0% in control cells, it increased to 7.4% for 30 min in the presence of the *A. lappa* ethanol extract at its minimal inhibitory concentration (MIC), 0.78 mg/ml, compared to uptake by heat-killed cells. Besides, leakage of DNA and proteins was observed in *A. lappa*-treated *C. albicans* cells. The increased uptake of EtBr and leakage of cellular materials suggest that *A. lappa* ethanol extract induced functional changes in *C. albicans* cell membranes. Incorporation of diphenylhexatriene (DPH) into membranes in the *A. lappa*-treated *C. albicans* cells at its MIC decreased to 84.8%, after 60 min of incubation, compared with that of the controls, indicate that there was a change in membrane dynamics. Moreover, the anticandidal effect of the *A. lappa* ethanol extract was enhanced at a growth temperature of 40°C compared to that at 35°C. The above data suggest that the antifungal activity of the *A. lappa* ethanol extract against *C. albicans* is associated with synergistic action of membrane permeabilization due to changes in membrane dynamics and cell wall damage caused by reduced formation of (1,3)-β-D-glucan and chitin.

## Introduction

*Candida* species are commensal yeasts that constitute normal human microbial flora of the skin, oral cavity, vagina, and gastrointestinal tract, but they are also the most common cause of opportunistic fungal pathogens in humans under conditions of incompetent host immunity [1]. *Candida albicans* is a principal cause of candidiasis. However, there has been a shift toward non-*albicans* *Candida* species; *C. tropicalis*, *C. glabrata*, *C. krusei*. *C. kefyr*, *C. parapsilosis*, *C. guilliermondii*, and *C. dubliniensis* are now commonly identified in *Candida* infections [2]. Despite intensive studies to explore new antifungal drugs, currently available drugs for treatment of candidiasis are restricted. Eukaryotic pathogens, including *Candida* species, share a close evolutionary relationship with their human hosts, which limits drug targets that can be utilized to kill the pathogen selectively [3]. There are four major classes of antifungals [4]: First, echinocandins change cell wall biosynthesis by inhibiting (1,3)-β-D-glucan synthase. Second, polyenes target the membrane by binding to ergosterol and promoting the formation of transmembrane channels, resulting in cellular ionic imbalance and cell death [5]. Third, azoles alter the cell membrane by inhibiting lanosterol-14α-demethylase for ergosterol biosynthesis [6]. Finally, fluoropyrimidines block the synthesis of fungal nucleic acids [7]. However, the emergence of multi-drug resistant *C. auris* [8], harmful side effects of currently available antifungals, and the ability of *Candida* to form biofilms all pose a serious health problem [9]. Thus, it is essential to expedite new effective antifungals, with new targets and inhibitory activity. Plant products are good candidates for these novel antifungals, since they have been used traditionally in ethnomedicine worldwide; many of these have been shown to have antimicrobial or antifungal activities able to treat various diseases [10]. In our previous research, we found that an ethanol extract from *A. lappa* has antifungal activity against *C. albicans*; the extract is associated with cell wall damage through inhibiting synthesis or assembly of both (1,3)-β-D-glucan polymers and chitin [11]. Here, we demonstrate that the *A. lappa* ethanol extract is also involved in changing membrane permeability.

## Materials and Methods

### Candida Strain

*C. albicans* SC5314, purchased from the American Type Culture Collection (USA), was used for the study.

### Preparation of the *A. lappa* Ethanol Extract

The *A. lappa* ethanol extract was prepared as described previously [11]. Briefly, dried *A. lappa* roots were soaked in 70% ethanol, concentrated, and lyophilized to obtain an *A. lappa* ethanol extract. The extract was dissolved in dimethyl sulfoxide (DMSO) to 100 mg/ml, filter-sterilized, and stored at -20°C until use.

### Calcofluor White-Propidium Iodide Dual Staining

*C. albicans* SC5314 cells (5 × 10^6^ cells/ml) were grown in YM media (10 g dextrose, 3 g malt extract, 5 g peptone and 3 g yeast extract in 1 liter of distilled water) in the presence of DMSO or 0.78 mg/ml of the *A. lappa* ethanol extract, incubated at 200 rpm and 37°C for 2.5 h. The cells were harvested, washed with phosphate-buffered saline (PBS, pH 7.4) (Invitrogen, USA), and stained with 10 μg/ml propidium iodide (PI) and 0.01% Calcofluor White M2R (Sigma, USA) in PBS (pH 7.4) for 5 min. Cells were examined using a bright-field microscope or a fluorescence microscope equipped with triple bandpass filters.

### Ethidium Bromide (EtBr)-Uptake Assay

The alteration of membrane permeability was investigated by EtBr uptake according to Rodrigues [12] with slight modifications. The uptake assay of EtBr was carried out using a fluorometer (Tecan, Austria). Exponential-phase *C. albicans* SC5314 cells were collected, washed with PBS (pH 7.4), and resuspended in PBS (pH 7.4) containing 1 μg/ml EtBr and 5 mM glucose to obtain a final cell density of 1 × 10^8^ cells/ml. Aliquots of 450 μl cell suspension were distributed into 5 microtubes, and DMSO or *A. lappa* ethanol extract was added at concentrations ranging from 0.39 to 1.56 mg/ml. Heat-killed cells were prepared by incubating the sample at 80°C for 10 min. One-hundred-μl aliquots of each sample were placed into a 96-well black/clear flat-bottom microplate (BD Falcon) in quadruplicate, and the foil-covered plate was incubated at 25°C. EtBr uptake in the *C. albicans* SC5314 cells was measured using a fluorometer, and fluorescence data were acquired at an excitation wavelength of 530 nm (bandwidth of 25 nm) and emission wavelength of 585 nm (bandwidth of 20 nm). Fluorescence intensity (arbitrary unit) of EtBr uptake in heat-killed cells was taken as 100, and uptake in cells treated with the *A. lappa* extract was represented as a percentage.

### Leakage of Cellular Materials

To determine whether the antifungal effect of the *A. lappa* ethanol extract is related to the permeability change in the *C. albicans* cell membrane, cellular leakage was assessed by fluorometric or spectrophotometric method. *C. albicans* cells in YM (1 × 10^9^ cells/ml) were incubated in the presence of the *A. lappa* ethanol extract (0.78 mg/ml) or the same volume of DMSO at 37°C at 200 rpm for 60 min. Then, *C. albicans* cells were washed twice with PBS (pH 7.4) by centrifugation at 12,000 ×*g* for 2 min, resuspended in PBS (pH 7.4), and further incubated at 37°C with agitation. At 20 min intervals, 0.6 ml cell suspension was removed and centrifuged at 12,000 ×*g* for 10 min, and the supernatant was saved for further analysis. The leakage of nucleotides was quantified using Qubit dsDNA BR (broad range) Assay Kit according to manufacturer instructions by Qubit 4.0 Fluorometer (Thermo Fisher, USA). To evaluate protein leakage, the absorbance of the supernatant (100 μl) mixed with Bradford reagent (Bio-Rad) was read at 595 nm using a microplate reader.

### Diphenylhexatriene (DPH) Incorporation into *C. albicans* Membranes

Log-phase *C. albicans* cells (5 × 10^8^ cells/ml) were incubated in the presence of DMSO or *A. lappa* ethanol extract at 37°C with agitation at 200 rpm. Every 30 min, a sample from each group of cell culture (0.9 ml) was centrifuged, washed with PBS (pH 7.4), and resuspended in PBS containing 50 μM DPH. Aliquots (0.2 ml) of each sample were distributed into a 96-well black/clear flat-bottom microplate in quadruplicate and further incubated at 25°C for 20 min in the dark on a rocker. Fluorescence was measured using a spectrofluorometer at 340 nm with a 20 nm bandwidth and 430 nm with a 20 nm bandwidth as excitation and emission wavelengths, respectively. The effect of *A. lappa* treatments compared with controls was analyzed using SigmaPlot 13.0. A *p* value less than 0.05 was regarded as statistically significant.

### Effect of Higher Temperature on Anticandidal Activity of *A. lappa* Extract

*C. albicans* cells were collected and the pellet was suspended in YM medium at A_595_ = 0.15. The cell suspension was mixed with DMSO or 0.39 mg/ml of *A. lappa* ethanol extract, and 200 μl aliquots of each sample were placed in quadruplicate in two 96-well flat-bottom microtiter plates. Then, each microplate was incubated with moist air at 35°C and 40°C, respectively, and cell growth was examined by measuring the absorbance of each culture at 595 nm every hour using a spectrofluorometer. Prior to measuring absorbance, microplates were agitated for 20 s.

### Changes in Electrolyte Concentration

Twenty ml of overnight *C. albicans* cells were harvested by centrifugation at 12,000 ×*g* for 10 min at 4°C and washed with cold PBS (pH 7.4). The cell pellet was suspended in 3 ml cold PBS, and the cell suspension was placed into 6 tubes. The *A. lappa* ethanol extract or DMSO corresponding to 0.5× minimal inhibitory concentration (MIC), 1× MIC, or 2× MIC was added, and tubes were incubated on ice with slight agitation on an orbital shaker. After 60 min, each cell suspension was harvested and washed twice with PBS (pH 7.4). Then, the pellet was resuspended in 0.5 ml PBS and incubated for an additional 30 min on ice with slight agitation. Subsequently, each cell suspension was centrifuged at 12,000 ×*g* at 4°C for 10 min, and the supernatant was saved for further analysis. The sodium, potassium, and chloride concentrations of each supernatant were measured using an electrolyte analyzer (SmartLyte, USA) after the instrument was calibrated using analytical grade sodium, potassium, and chloride QC standards. The sodium and potassium data are shown.

## Results and Discussion

Our earlier studies on *A. lappa* ethanol extract demonstrated that the extract has antifungal activity against *Candida* species, and the MIC of the *A. lappa* ethanol extract against *C. albicans* SC5314 was found to be 0.78 mg/ml [11] as follows. Chitin content was reduced to 79.8% relative to control after incubation with *A. lappa* ethanol extract at its MIC for 6 h. Synthesis of (1,3)-β-D-glucan polymers was inhibited to 84.3% of control treatment following incubation of *C. albicans* microsomes with the *A. lappa* extract at its MIC. This indicates that *A. lappa*-treated cells with weakened cell walls are more sensitive to osmotic pressure. In fact, *A. lappa*-treated *C. albicans* cells survived in the presence of 0.8 M sorbitol, as demonstrated by the fact that *A. lappa* extract MIC against *C. albicans* cells demonstrated an eight-fold increase from 0.78 mg/ml to 6.24 mg/ml in 3 days [11]. The present study was designed to show that *A. lappa* ethanol extract is also involved in membrane damage in *C. albicans* cells.

### Dual Staining with Calcofluor White and PI

**Fig. 1:**
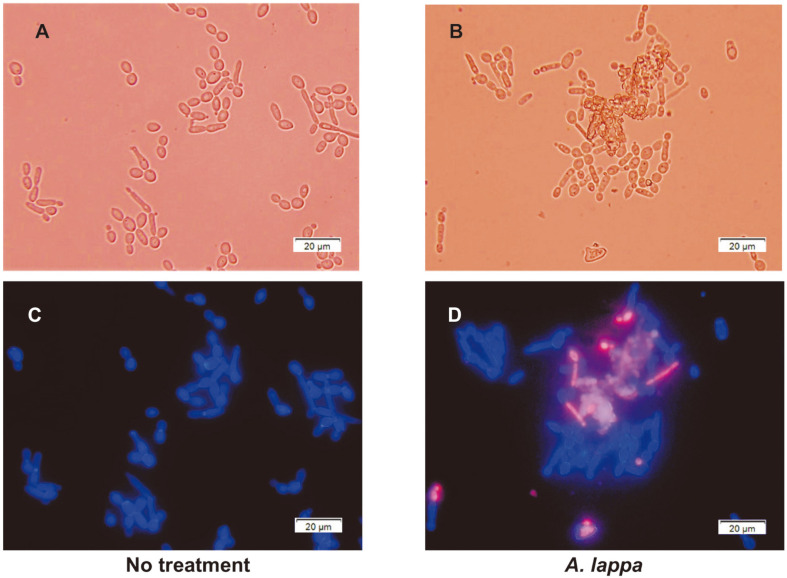
Membrane-damaged *C. albicans* cells shown by Calcofluor White-PI dual staining. *C. albicans* cells treated with DMSO (**A and C**) or the *A. lappa* ethanol extract (**B and D**) for 2.5 h were stained with both 10 µg/ml PI and 0.01%Calcofluor White, and observed with a bright-field microscope (**A and B**) or a fluorescence microscope equipped with triple bandpass filters. The *Candida* cell walls were stained blue with Calcofluor White (**C and D**), and cells with damaged membranes were stained pink with PI (**D**). Beige aggregates are the cell clusters with severely injured cell membranes in the center (**D**).

Calcofluor White, a fluorescent dye that appears blue under ultraviolet light, stains the chitin layer in the cell walls of fungi [13]. PI, a red fluorescent dye, is a cationic molecule that is able to pass through a damaged cell membrane, but is unable to enter into cells with an intact cell membrane. Consequently, dead or dying cells with defective cell membranes are characterized by red fluorescence signals, while those with intact cell membranes are not stained with PI [14]. *C. albicans* SC5314 cells treated with DMSO or ethanol extract of *A. lappa* at 37°C for 2.5 h were stained with Calcofluor White and PI, then observed under bright-field and fluorescence microscopy. In contrast to blue cells with intact cell membranes in the controls (Fig. 1C), some pink cells were found among *A. lappa*-treated cells, indicating that these cells were sick or dead with damaged cell membranes (Fig. 1D). Moreover, clumps with beige fluorescence seemed to be dead cell aggregates with severely injured membranes without rupture (Fig. 1D). Aggregate formation may occur through cooperation with individual cells, since cell aggregation is known to be stimulated by cell adhesion molecules, secreted enzymes, and molecules that promote quorum sensing in yeast [15], which is a virulence factor for fungal adhesion and colonization [16]. Aggregate formation in *A. lappa*-treated cells is assumed to be a protective response against harsh conditions imposed by the *A. lappa* ethanol extract. Based on data from PI staining, we examined whether *A. lappa* ethanol extract affects the function of *C. albicans* membranes by EtBr-uptake assay.

### EtBr-Uptake Assay

**Fig. 2:**
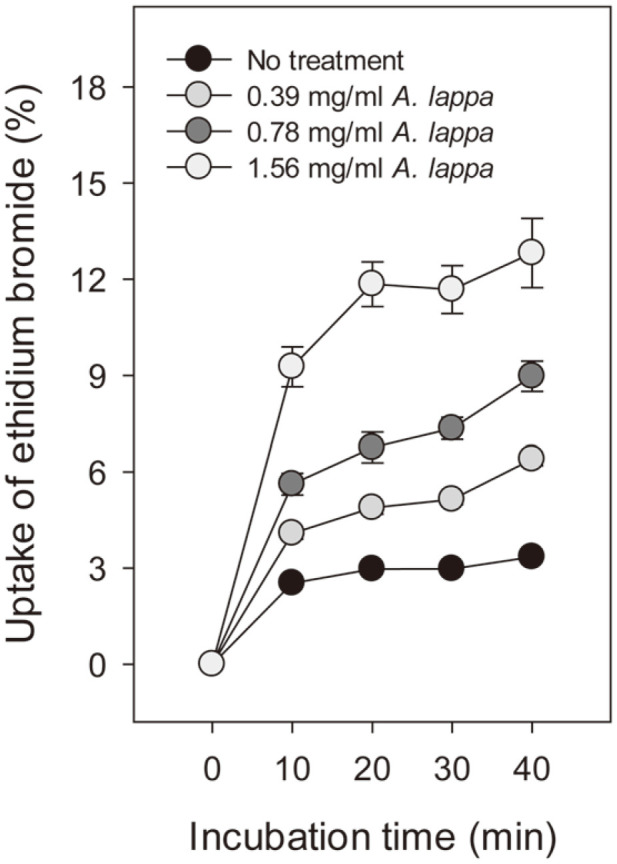
Uptake of ethidium bromide. *C. albicans* cells in PBS (pH 7.4) containing 1 µg/ml EtBr and 5 mM glucose were incubated with DMSO or the *A. lappa* ethanol extract. Heat-killed cells were included as a positive control. Amount of EtBr uptake by *C. albicans* cells was estimated using a fluorometer, and fluorescence intensity of the heat-killed cells was taken as 100. Three independent experiments were performed and the data represent the mean and standard deviations from a representative experiment.

The fluorescent probe EtBr is an intercalating agent resembling a DNA base pair. The molecule emits weak fluorescence in aqueous solution, but exhibits a 20- to 25-fold fluorescence enhancement upon binding to double-stranded DNA [17]. Due to its charged and polar nature, EtBr cannot penetrate intact membranes. While the molecule is able to enter damaged cells rapidly, where it binds mainly to DNA, the dye is mostly excluded by intact cells [18]. Therefore, EtBr is commonly used to indicate cell membrane integrity. In our preliminary experiment, *C. albicans* cells incubated with 40 μg/ml EtBr for 30 min were visualized by fluorescence microscopy, and a considerable degree of orange fluorescence and weak orange fluorescence were detected in the nucleus and the cytoplasm, respectively, irrespective of treatment with *A. lappa* ethanol extract (Fig. S1). In contrast to the previous report, a high concentration of EtBr is thought to penetrate cell membranes by passive transport or other mechanisms. In addition, it is thought that EtBr binds double-stranded DNA strongly in the nucleus and base-paired RNA or DNA-RNA hybrid weakly in the cytoplasm. Nonetheless, the *A. lappa*-treated cells appeared to fluoresce orange more intensely in both the nucleus and cytoplasm relative to controls. These results suggest that *A. lappa* ethanol extract is involved in damaging *C. albicans* cell membranes. To explore these findings further, the experiment was modified to quantify membrane damage using a fluorometer. In the EtBr-uptake assay, *C. albicans* cells in PBS containing 5 mM glucose and 1 μg/ml EtBr were incubated with DMSO or the *A. lappa* ethanol extract at 25°C. Glucose was added to the buffer to promote efflux system activity. Fluorescence intensity of EtBr (in AU, arbitrary units) in *A. lappa*-treated *C. albicans* cells was represented as a percentage, while that of heat-killed cells was set as 100. Uptake of EtBr by DMSO-treated controls was 2.5 ± 0.1% compared to heat-killed cells at 10 min (Fig. 2). Uptake of EtBr by *A. lappa*-treated cells at 0.39 mg/ml, 0.78 mg/ml, and 1.56 mg/ml was 4.1 ± 0.2%, 5.6 ± 0.3%, and 9.3 ± 0.6% at 10 min, respectively, relative to heat-killed cells. There was a statistically significant difference between *A. lappa*-treated or heat-treated and DMSO control group (*p* < 0.001). Furthermore, *A. lappa*-treated cells showed significant uptake of EtBr in *C. albicans* cells after 20, 30, and 40 min compared to controls (*p* < 0.001). As shown in Fig. 2, EtBr uptake by *A. lappa*-treated cells increased slowly in a time- and concentration-dependent manner. Thus, it appears that more EtBr molecules easily enters across the damaged cell membranes of the *A. lappa*-treated *C. albicans* cells and intercalate into DNA or DNA-RNA hybrid gradually. The data demonstrate that *A. lappa* ethanol extract brings about permeabilization in *C. albicans* membranes and support those of PI staining (Fig. 1D).

### Leakage of Cellular Materials

**Fig. 3:**
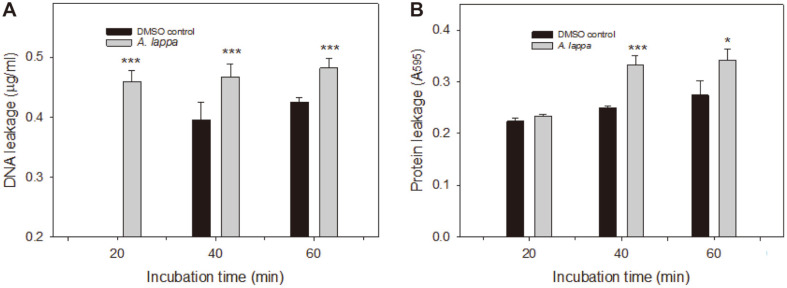
Leakage of cellular materials. *C. albicans* cells treated with DMSO or *A. lappa* ethanol extract were washed with PBS (pH 7.4) twice, and incubated further as indicated. Then, each cell suspension was centrifuged and the supernatant was analyzed for (**A**) the leakage of DNA and (**B**) protein leakage, the absorbance at 595 nm of the supernatant with Bradford reagent. The data represent average values and standard deviations obtained in three independent experiments. *p < 0.05, ***p < 0.001

UV absorbance spectrophotometry is a commonly used method for quantification of nucleic acids, since DNA and RNA have high absorbance peaks at 260 nm. Initially, we used the spectrometric method, but it was so delicate that the nucleotide data were not stable. Therefore, the leakage of nucleotides was estimated using Qubit dsDNA BR Assay Kit, which is based on fluorescence, and used for the quantification of double-strand DNA samples. As shown in Fig. 3, both DNA and proteins were detected in the extracellular buffer of the *A. lappa* extract-treated cells. It is surprising that 0.396 ± 0.018 μg/ml DNA was detected in the extracellular buffer of the DMSO control cells at 40 min, although no detectable amount of DNA was found at 20 min. Nevertheless, the amount of leaked DNA was higher in the extracellular buffer of the *A. lappa*-treated cells at 20, 40, and 60 min, respectively, compared with that of the DMSO controls (Fig. 3A). There was a statistically significant difference between the control and each *A. lappa*-treated group (*p* < 0.001).

The Bradford assay was carried out for protein quantification [19] according to the manufacturer’s instructions (Bio-Rad). Proteins were detected in the extracellular buffer of both the *A. lappa*-treated cells and the DMSO controls, too. Since the Bradford assay is a colorimetric assay, it was expected that the extracellular buffer of the *A. lappa*-treated cells contains much more proteins judged by the deeper-blue color of the samples. However, the difference of the extracellular buffer between the *A. lappa*-treated cells and DMSO controls was not statistically significant at 20 min. There was a statistically significant difference between the control and *A. lappa*-treated groups after 40 min (*p* < 0.05). The reason why leakage of DNA and proteins were found in the extracellular buffer of the DMSO controls is assumed to be due to the presence of 0.78% DMSO, which is a solvent for the ethanol extract of *A. lappa*, and high concentration of *C. albicans* (1 × 10^9^ cells/ml) in the assay. In our preliminary experiment, more than 1% DMSO showed toxic effects on red blood cells. Regardless of the effect of DMSO, it is certain that the *A. lappa* ethanol extract made cellular leakage effects on *C. albicans* cells.

### DPH Intercalation into *C. albicans* Cell Membranes

**Fig. 4:**
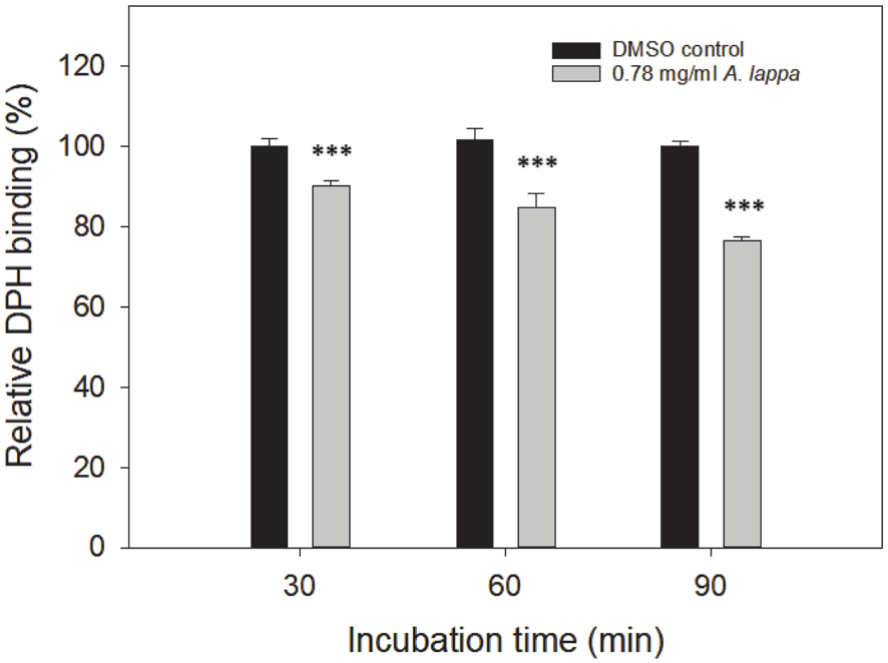
DPH intercalation into *C. albicans* cell membranes. *C. albicans* cells treated with DMSO or the *A. lappa* ethanol extract at 37°C were resuspended in PBS (pH 7.4) containing 50 µM DPH. Then, fluorescence of each sample was measured using a spectrofluorometer. The data represent average values and standard deviations obtained in three independent experiments. ***p < 0.001.

DPH is a membrane probe commonly used to study fluorescence anisotropy; rotational motion of DPH is monitored to investigate membrane fluidity and lipid ordering [20]. Whether changes in membrane permeability are related to the lipid composition or arrangement of *C. albicans* cell membranes was assessed, and DPH intercalation into the membranes of DMSO controls and *A. lappa*-treated cells was compared. DPH is not fluorescent in water but strongly fluorescent after intercalation into lipid membranes. When added to the DMSO-treated or the *A. lappa*-treated *C. albicans* cells, DPH intercalated within the acyl chain region of the lipid bilayer, but any differences in fluorescence intensity between treatment groups are related to changes in the arrangement or composition of acyl chains. Relative intercalation of DPH into the *A. lappa*-treated cells at its MIC was 90.3 ± 1.2%, 84.8 ± 3.5%, and 76.5 ± 0.8% after 30, 60, and 90 min of incubation, respectively, compared with that of the DMSO controls (Fig. 4). There was a statistically significant difference between the control and *A. lappa*-treated groups (*p* < 0.001). Decreased intercalation of DPH into *A. lappa*-treated *C. albicans* membranes suggests that there was a change in membrane dynamics due to the alteration of lipid arrangement or composition. Increased membrane fluidity is related to a high density of fatty acids with short chains, unsaturated fatty acids, or low density of ergosterol. When the extract was examined for effects on ergosterol synthesis, no significant effect was detected (data not shown).

### Effect of Higher Temperature on Anticandidal Activity of *A. lappa* Extract

**Fig. 5:**
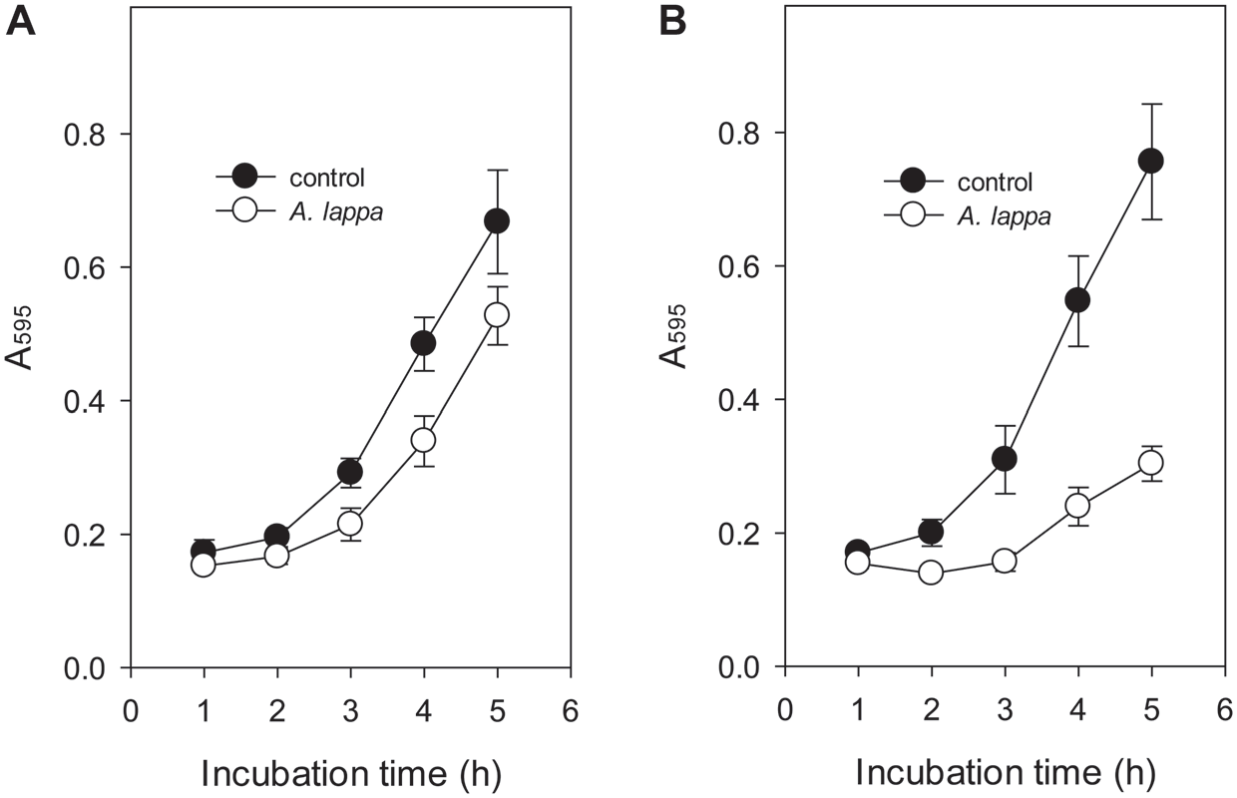
Effect of higher temperature on anticandidal activity of the *A. lappa* extract. Exponential-phase *C. albicans* cells (A_595_ = 0.15) were incubated with DMSO or the *A. lappa* ethanol extract at 35°C (**A**) and 40°C (**B**), respectively. Every hour, cell growth was checked by measuring the absorbance of each sample at 595 nm. Data represent average values and standard deviations obtained in two independent experiments.

The growth of *C. albicans* cells at 35°C and 40°C in the presence of DMSO or *A. lappa* ethanol extract at 0.39 mg/ml was compared by measuring the absorbance at 595 nm hourly (Fig. 5). DMSO control cells grew faster at 40°C than at 35°C, while *A. lappa*-treated cells grew more slowly at 40°C than at 35°C. That is, the *A. lappa* ethanol extract showed better antifungal activity at higher temperatures. We can assume that the anticandidal effect of *A. lappa* ethanol extract against *C. albicans* is enhanced because of the synergistic effect of increased membrane fluidity at higher temperatures with the membrane perturbation caused by the extract.

### Changes in Electrolyte Concentration

**Fig. 6:**
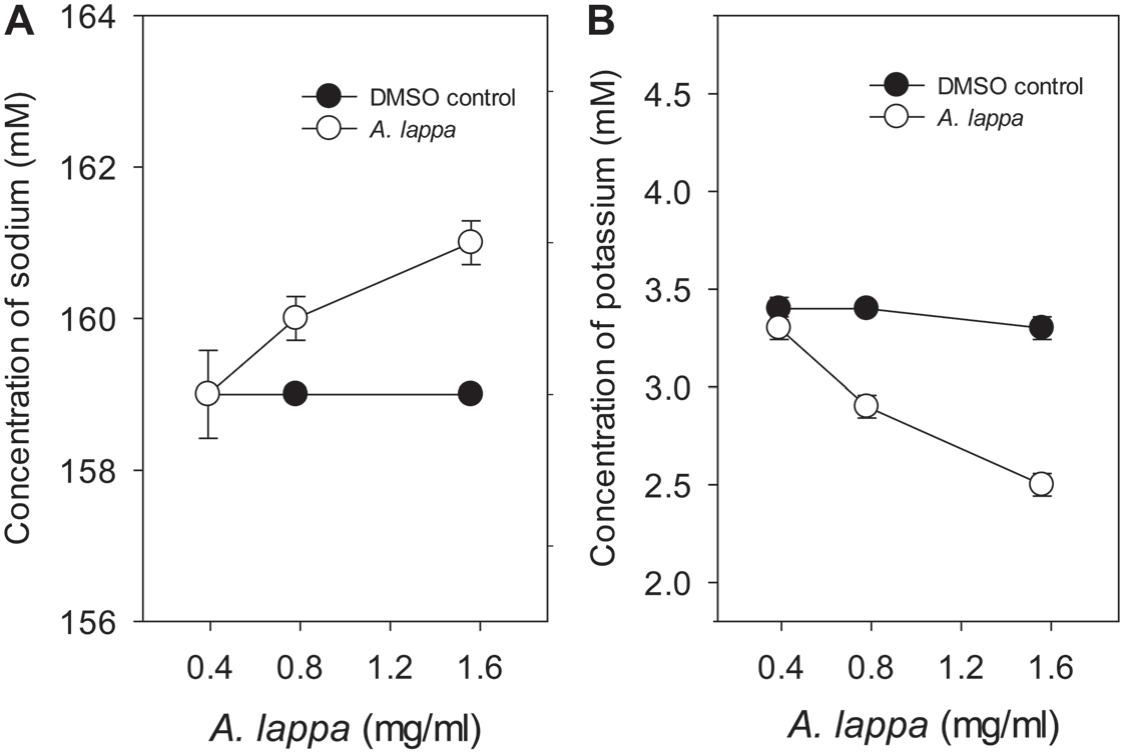
Changes in electrolyte concentration of *C. albicans*. Overnight cultures of *C. albicans* cells (50 ml) were suspended in cold PBS (pH 7.4) and incubated in the presence of DMSO or *A. lappa* ethanol extract for 60 min. Each cell culture was harvested, resuspended in PBS (pH 7.4), and incubated for an additional 30 min. Then, each sample was centrifuged, and the supernatant was subjected to electrolyte analysis. Sodium and potassium concentrations of each supernatant were measured using an electrolyte analyzer; these data represent one of the results from three repeated experiments.

*C. albicans* cells treated with DMSO or *A. lappa* ethanol extract for 60 min were washed, and cells were incubated for an additional 30 min. Cells were then harvested, and the supernatant was saved to evaluate sodium, potassium, and chloride concentrations using an electrolyte analyzer. When *C. albicans* cells were treated with 0.5× MIC (0.39 mg/ml) of *A. lappa* ethanol extract, the sodium concentration of the supernatant was 159 mM, the same as that of the DMSO control (Fig. 6A). However, when *C. albicans* cells were incubated with 1× MIC or 2× MIC of *A. lappa* ethanol extract, the sodium concentration of the extracellular supernatant increased from 159 mM to 160 mM and 161 mM, respectively, whereas that of the DMSO control cells remained the same at 159 mM (Fig. 6A). In contrast, when *C. albicans* cells were exposed to 2× MIC of *A. lappa* ethanol extract, the potassium concentration of the supernatant decreased from 3.3 mM to 2.5 mM, although that of the DMSO control remained stable at approximately 3.4 mM (Fig. 6B). That is, 2 mM sodium ion flowed out and 0.8 mM potassium ion flowed in following treatment with *A. lappa* extract at 1.56 mg/ml for 60 min. However, the extracellular chloride concentration of the DMSO control and *A. lappa*-treated *C. albicans* cells remained the same at 151 mM (data not shown). Potassium is essential for many cellular functions, including regulation of cell volume and intracellular pH, maintenance of stable membrane potential, compensation of negative charges in many macromolecules, protein synthesis, and enzyme activation [21]. Potassium can be highly accumulated in different types of living cells due to its essential role in many physiological functions. In contrast to the essential function of potassium ions, high concentrations of sodium are toxic to cells. Therefore, yeast cells maintain an optimum intracellular concentration of potassium ions and a high intracellular K+/Na+ ratio with three different strategies: transporters display higher affinity for potassium than for sodium, toxic or surplus cations undergo efficient efflux from cells, and organelles selectively sequester cations [21].

On the basis of the increased uptake of EtBr data, we conclude that *A. lappa* ethanol extract generated functional changes in *C. albicans* cell membranes. In particular, *A. lappa*-treated *C. albicans* cells seemed to be damaged in membrane structure. In terms of membrane permeabilization, DNA and proteins were found in the extracellular buffer of *A. lappa*-treated *C. albicans* cells. Reduction of DPH intercalation into *A. lappa*-treated cells indicates alterations in lipid composition or arrangement, followed by increased membrane permeability in *C. albicans* cells. The enhanced antifungal activity of *A. lappa* extract at the elevated temperature of 40°C supports the hypothesis that membrane permeability by the extract is amplified at higher temperatures. Perturbation of cell membranes, leakage of proteins or salts, or influx of salts are involved in destabilizing the osmotic balance of *C. albicans* cells. Changes in sodium and potassium ion concentrations occurred in the extracellular buffer of *A. lappa*-treated cells, possibly restoring osmotic balance in response to membrane permeabilization. Nevertheless, cell aggregates were frequently formed by *A. lappa* treatment. The aggregates might be formed by membrane-membrane fusion among individual cells with weakened cell walls. Aggregate formation could be a protective response for adhesion for colonization, or it may be for switch from the yeast form to the hyphal form.

In conclusion, the antifungal effect of *A. lappa* ethanol extract is related to the synergistic action of the inhibition of cell wall synthesis and increased membrane permeability in *C. albicans*.

## Supplemental Material



Supplementary data for this paper are available on-line only at http://jmb.or.kr.
