# Supplementary material for: Aucklandia lappa Causes Membrane Permeation of Candida albicans
Source: J Microbiol Biotechnol. 2020 Nov 4;30(12):1827–34. doi: 10.4014/jmb.2009.09044 (PMC9728268; doi:10.4014/jmb.2009.09044)

J. Microbiol. Biotechnol.
https://doi.org/10.4014/jmb.2009.09044

Figure S1. Uptake of ethidium bromide.

C. albicans cells in PBS (pH 7.4) containing 40 µg/mL EtBr were incubated with DMSO (A and C) or 0.78 mg/ml of the *A. lappa* ethanol extract (B and D) for 30 min. Cells were observed with a bright-field microscope (A and B) or a fluorescence microscope (C and D). A detectable and weak orange fluorescence were observed in the nucleus and the cytoplasm, respectively, irrespective of treatment with *A. lappa* ethanol extract.

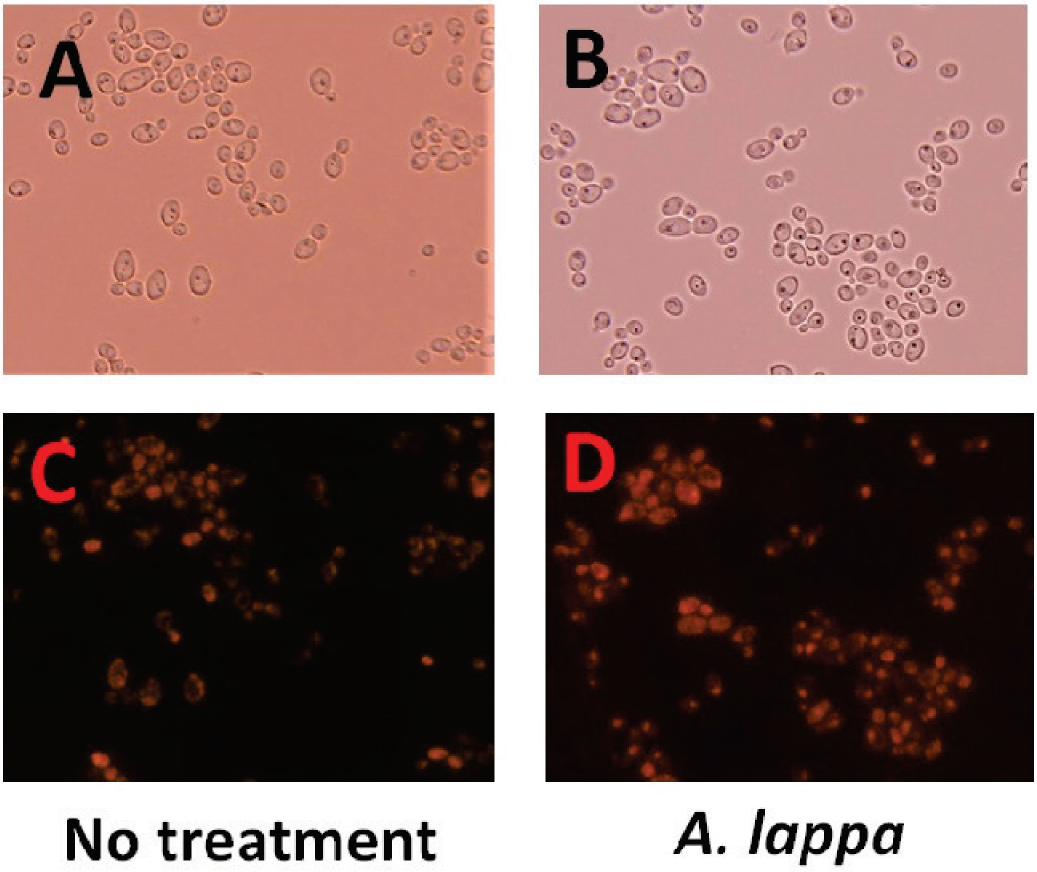

Supplement: Supplementary file 1 [file JMB-30-12-1827-supple.pdf]
